# Tracing CO_2_ sources in urban and rural areas characterized by different land-use patterns using carbon isotopes

**DOI:** 10.1371/journal.pone.0326306

**Published:** 2025-06-17

**Authors:** Eui-Kuk Jeong, Youn-Young Jung, Seung-Hyun Choi, Moojin Choi, Kwang-Sik Lee, Woo-Jin Shin

**Affiliations:** 1 Research Center of Earth and Environmental Sciences, Korea Basic Science Institute, Cheongju-si, Chungbuk, Republic of Korea; 2 Graduate School of Analytical Science and Technology, Chungnam National University, Daejeon, Republic of Korea; University of Maryland Center for Environmental Science, UNITED STATES OF AMERICA

## Abstract

Air samples were collected from urban and rural areas of Korea with different land-use patterns between October 2022 and April 2023 to identify the sources of atmospheric CO_2_. We analyzed representative end-members from natural and anthropogenic sources (soil, vehicle exhaust gases, and coal) to determine the CO_2_ concentrations and carbon isotope compositions of CO_2_ (δ^13^C-CO_2_). Urban samples exhibited lower δ^13^C values and higher CO_2_ concentrations than rural samples. Both urban and tunnel samples showing similar slopes and intercepts on the plot of 1000/CO_2_ vs. δ^13^C-CO_2_ likely shifted toward the vehicle exhaust end-member. Among the rural samples, those collected from coastal areas showed trends similar to the urban and tunnel samples, which differed from those collected from inland areas. This suggests that samples from coastal areas were affected by CO_2_ emissions from coal-fired power plants in the region. In contrast, inland samples showed the highest slope and lowest intercept (i.e., estimated δ^13^C-CO_2_ value of −27.0‰), suggesting that natural sources such as soil CO_2_ dominate the contribution to atmospheric CO_2_ in inland areas. This study demonstrates that the primary CO_2_ sources in regions with different land-use patterns can be distinguished using the relationship between atmospheric CO_2_ concentrations and δ^13^C-CO_2_ values.

## Introduction

Carbon dioxide (CO_2_) is a major greenhouse gas closely associated with global warming [1]. The CO_2_ concentration on Earth has rapidly increased since the pre-industrial period, from 280 ppm in the 1700s to 414 ppm in 2020 [2]. Increased CO_2_ concentrations are regarded as a major cause of climate change and extreme weather [2–4]. Thus, many countries worldwide have tried to reduce the amount of CO_2_ released from fossil fuels [5]. In line with this global issue, identifying the CO_2_ sources in the atmosphere has also been studied for a long time [6–9].

Atmospheric CO_2_ concentration is predominantly controlled by the input of fossil fuel (gasoline, diesel, and coal) combustion and soil CO_2_ contribution from photosynthesis-respiration processes [10–12]. Identifying the source of atmospheric CO_2_ can be challenging because of the complicated CO_2_ mixing processes that occur between the hydrosphere, pedosphere, biosphere, and atmosphere [13–16], as well as the production and consumption of CO_2_ within these spheres [17–19]. For example, activities such as electricity generation, transportation, and urbanization are representative sources of anthropogenic CO_2_ emissions and their contributions to atmospheric CO_2_ would vary spatiotemporally depending on factors affecting CO_2_ concentrations; i.e., population density, electricity production, and transportation intensity are locally different. Additionally, local land-use patterns can affect the consumption or production of both natural and anthropogenic CO_2_, leading to regionally variable contributions to atmospheric CO_2_ levels.

The major CO_2_ sources varied in their concentrations and carbon isotopic compositions. Carbon dioxide emitted from fossil fuel combustion typically shows CO_2_ concentrations from 1 to 15% and δ^13^C-CO_2_ values from –40.5 to –22.8‰ [20–25]. In areas where C3 plants prevail extensively, soil CO_2_ concentrations range from several thousand ppm to tens of thousands, and δ^13^C-CO_2_ values ranges from –30 to –23‰ [26,27]. In specific environments such as waste landfill, CO_2_ has relatively enriched ^13^C unlike the δ^13^C values of typical soil CO_2_. Organic matter is naturally decomposed in the landfill and produces ^13^C-depleted biogenic methane gas. The resulting CH_4_ typically has δ^13^C values from −65 to −50‰, and the residual CO_2_ has relatively high δ^13^C-CO_2_ value from −15.9 to 0.7‰ [28,29]. In previous studies, the δ^13^C-CO_2_ values of fumarole samples ranged from −0.95 to −2‰ (average −1.48 ± 0.22‰, n = 78), having CO_2_ concentrations reaching up to 36,000 ppmv [30,31]. In area with continuous degassing and seismic activity, the emitted CO_2_ had a δ^13^C value of −1.67‰ [32]. Identifying the CO_2_ source in the atmosphere using only one parameter or a simple correlation is difficult. Therefore, many studies have used the relationship between 1/CO_2_ concentration and δ^13^C-CO_2_ value, known as the Keeling plot, to trace the origin of CO_2_ sources in the atmosphere. Typically, the binary mixing line generated from a Keeling plot represents the relationship between atmospheric CO_2_ (i.e., tropospheric CO_2_) and another CO_2_ source (e.g., contaminant sources). This relationship is expressed by the equation: δ^13^C-CO_2_ = a × 1/CO_2 _+ b. Widory and Javoy [22] reported that CO_2_ gases exhibited different slopes and intercepts in polluted and unpolluted air in Paris, suggesting that fossil fuel combustion and human respiration influence the urban atmosphere. Clark-Thorne and Yapp [7] found that urban and rural samples exhibited estimated lines with different slopes and intercepts on a Keeling plot. Furthermore, seasonal variations in winter and summer were observed along the slope and intercept owing to the contribution of soil CO_2_ to the atmosphere. Recently, the effects of urbanization on atmospheric CO_2_ concentrations and δ^13^C values have been reported using Keeling plots [33].

This study aims to determine the primary sources of atmospheric CO_2_ in South Korea. To this end, we analyzed the CO_2_ concentrations and carbon isotope compositions of representative natural (soil CO_2_) and anthropogenic (vehicle exhaust gases and coal combustion gas) sources. In addition, we collected atmospheric CO_2_ from urban and rural areas to evaluate whether CO_2_ emissions originated from different sources as urbanization increased. Rural areas were subdivided into regions with coal-fired power plants and forest-dominated areas. Keeling plots were used to identify CO_2_ sources in the study area. The approach used to interpret atmospheric CO_2_ sources in this study can assess the contribution of CO_2_ from natural and anthropogenic sources to the atmosphere.

### Study area

#### Urban area.

Seoul, the capital of South Korea, has an area of 605.2 km² and is a densely populated city, with approximately 20% (9 million people) of the country’s total population [34]. According to the latest statistics on land-use patterns in Seoul [35], the city consists of residential, business, and public facilities (40.9%); forests and open spaces (26.7%); transportation facilities (14.5%); and surface water (8.8%) (Table 1). The transportation facility area includes 55 vehicular tunnels throughout the city. Fifteen tunnels are over 1 km in length. Approximately 3.1 million vehicles are registered in the city, accounting for 12% of the country’s total. According to the 2022 Seoul Transportation Statistics [36], the city’s traffic volume is approximately 17 million vehicles (including public buses) daily.

**Table 1:** Statistics on the land-use patterns in the administrative districts where air samples were collected in this study.

Land-use patterns	Seoul	Chungju	Seosan	Dangjin	Taean
	(10^3^ m^2^)				
Dry paddy	9,205	78,226	78,152	70,007	64,509
	(1.5)	(8.0)	(10.5)	(9.9)	(12.5)
Rice paddy	7,833	77,188	190,781	209,386	108,773
	(1.3)	(7.8)	(25.7)	(29.7)	(21.1)
Forest field	137,416	612,576	285,925	225,841	231,361
	(22.7)	(62.3)	(38.5)	(32.0)	(44.8)
Building land	222,884	27,240	23,661	24,658	15,190
	(36.8)	(2.8)	(3.2)	(3.5)	(2.9)
Factory site	2,849	10,756	19,056	23,827	3,027
	(0.5)	(1.1)	(2.6)	(3.4)	(0.6)
School	24,871	2,868	1,344	1,730	855
	(4.1)	(0.3)	(0.2)	(0.2)	(0.2)
Road	80,505	39,210	27,107	36,519	17,362
	(13.3)	(4.0)	(3.7)	(5.2)	(3.4)
Railway	7,104	1,406	0	0	0
	(1.2)	(0.1)	(0)	(0)	(0)
River	52,050	37,962	9,278	9,433	1,621
	(8.6)	(3.9)	(1.2)	(1.3)	(0.3)
Ditch	3,255	14,410	19,704	28,815	12,775
	(0.5)	(1.5)	(2.7)	(4.1)	(2.5)
Marsh	1,510	32,157	42,616	39,986	23,636
	(0.2)	(3.3)	(5.7)	(5.7)	(4.6)
Park	23,972	1,520	2,157	3,191	293
	(4.0)	(0.2)	(0.3)	(0.5)	(0.1)
Miscellaneous area	16,292	16,137	22,664	12,444	14,978
	(2.7)	(1.6)	(3.1)	(1.8)	(2.9)
Total	605,208	983,674	742,284	705,532	515,979
	(100)	(100)	(100)	(100)	(100)

The number in the parenthesis represents the proportion of land-use patterns in each administrative district. The statistics are from the Ministry of Land, Infrastructure and Transport (2023).

#### Rural areas.

Air samples from rural areas were collected from three locations along the western coast (Dangjin, Seosan, and Taean) and an inland area (Chungju). The areas of Dangjin, Seosan, Taean, and Chungju are 705 km², 740 km², 516 km², and 984 km², respectively. The four sampling regions have significantly lower populations, corresponding to <2% of Seoul’s population; Dangjin, Seosan, Taean, and Chungju represent residents of 160,000, 170,000, 60,000, and 200,000 populations, respectively [34]. All regions have the highest proportion of forests (32–62%), followed by agricultural areas (18–41%), surface water (5–7%), and residential and business areas (3–4%) (Table 1) [35]. The number of registered vehicles in each rural area ranged from 37,000–120,000, less than 4% of that in Seoul [37]. In addition, there are 38 fishing ports and 3 harbors along the western coast [38]. In Dangjin and Taean regions, coal-fired power plants are located near these harbors and supply approximately 40% of the country’s electricity [39].

## Methods

### Ambient air samples

**Fig 1:**
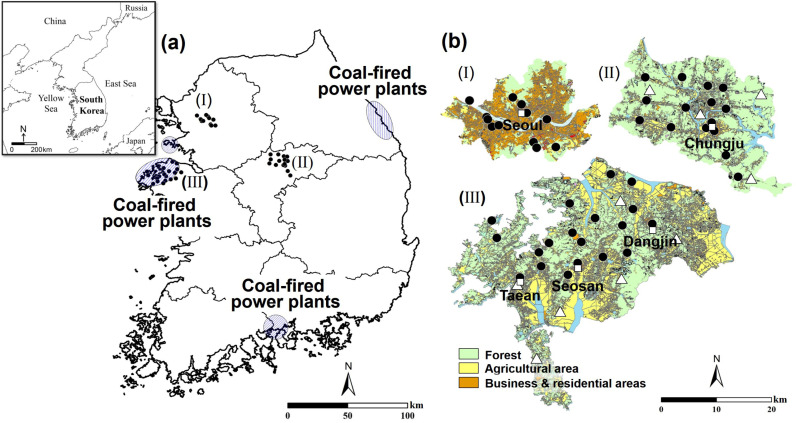
Maps showing locations where air and coal samples were collected in South Korea. The locations of coal-fired power plants are denoted by blue circles with diagonal lines (a). The black symbols in (Ⅰ), (Ⅱ), and (Ⅲ) represent the air sampling sites in Seoul, Chungju, and West coast regions, respectively, and the white triangle symbols in (Ⅱ) and (Ⅲ) indicate soil sampling sites (b).

Ambient air samples were collected from the urban (Seoul) and rural (suburban) areas between October 2022 and April 2023 (Fig 1). Of the samples collected from the urban area (n = 21), 12 were collected from six tunnels with lengths of over 1 km. All tunnels, whether straight or curved, have two lanes in each direction and jet fan ventilation systems are installed overhead to circulate air inside. Except for one sample collected from the Hongjimoon Tunnel, the remaining tunnel samples were collected during rush hour to measure CO_2_ concentrations and isotopic compositions in ambient air predominantly influenced by vehicle exhaust. All air samples from tunnels were collected while driving in both directions, by pumping ambient air through vehicle window into Tedlar bags. Other urban samples were mostly collected from the roadside or through vehicle windows while driving; two were collected from a building approximately 20 m high in the city. Rural samples were collected near the western coastline in Korea, where coal-fired power plants are located, and from the central Korean region, where forested areas are relatively dominant. The sampling was conducted in sparsely populated areas. The former was collected monthly from October 2022 to February 2023, whereas the latter was collected in April 2023. Inland areas were sampled twice, in February and April 2023. Additionally, ambient air samples were collected from forested areas to estimate the background CO_2_ concentrations and isotopic compositions. The sampling campaign was conducted four times, on October 22, 2022, and January 7, 2023, in the middle of a mountain at 229 m above sea level. All samples, with an exception for tunnel samples, were collected before sunset to minimize the diurnal variation of CO_2_ level caused by both natural (e.g., photosynthesis and respiration) and anthropogenic (e.g., vehicles) sources during the study period. All air samples were collected approximately 2 m above ground level, passed through a dust filter and moisture absorbent, and then transferred to a 10-L Tedlar bag with a stopcock (polyvinyl chloride, SL.Bag3505) connected to a pump operating at a flow rate of 200 ml/min for 20 min. The bag was rinsed with 99.999% nitrogen gas three times in the laboratory before doing sampling campaign and it was reused during this study period. The tunnel samples were collected while driving through both sides of the tunnel. For air samples in sample bags, carbon isotopic compositions (δ^13^C) and CO_2_ concentrations were analyzed using a Picarro G2131-*i* Analyzer (Picarro, Santa Clara, CA, USA) at the Korea Basic Science Institute (KBSI). Two standard materials with different CO_2_ concentrations and carbon isotope compositions (cylinder #CC707290: 411.3 ppm for CO_2_ and −8.673‰ for δ^13^C; and cylinder #CC702380: 435.38 ppm for CO_2_ and −10.065‰ for δ^13^C) were used to generate a calibration line with an estimated slope and offset (less than 0.5 ppm for CO_2_ concentration-drift test). These standard materials were calibrated by the Stable Isotope Lab at INSTAAR (University of Colorado), in cooperation with the Global Monitoring Division of the National Oceanic and Atmospheric Administration (NOAA). δ^13^C values were expressed relative to the V-PDB standard, using delta (δ) notation: δ^13^C (‰) = [(R_sample_/R_reference_) – 1] × 1000, where R represents ^13^C/^12^C ratio. According to the V-PDB scale, the reference ^13^C/^12^C ratio (R_reference_) is internationally accepted as 0.011802 [40], the ^13^C/^12^C ratio of the samples (R_sample_) was calculated for the two standard materials, and the slope and offset were determined. The measurements were conducted for approximately 60 min per bag, and the analyzed data were averaged.

### Vehicle exhaust gas and coal samples

Vehicle exhaust gas, a major anthropogenic source of atmospheric pollution, was collected directly from the tailpipes of several types of vehicles (two gasoline and three diesel vehicles; Table 2). The vehicles used in the experiment were driven until the temperature gauge stabilized before the exhaust gas was collected. When the vehicle was idling, a nylon tube with an inner diameter (ID) of 3.21 mm and an outer diameter (OD) of 6.35 mm was inserted into the tailpipe, and the other end of the tube was connected to a 47-mm PTFE dust filter with a pore size of 1.0 μm. Then, exhaust gas samples were collected in a 10-L Tedlar bag through a dust filter and a water trap using a pump at a flow rate of 200 ml/min for 20 min. Because the CO_2_ concentration was extremely high, the exhaust gas was mixed with 99.999% nitrogen gas in a separate sampling bag to control CO_2_ concentration in the 400–1000 ppm range. The carbon isotope composition of the mixed gas was determined using a Picarro G2131-*i* Analyzer (Picarro, Santa Clara, CA, USA) at KBSI in the same manner. Coal samples imported to South Korea from eight countries (Canada, Russia, Australia, the USA, Philippines, Indonesia, South Africa, and Colombia) were collected from major coal-fired power plants in South Korea [41]. The preparation and analysis of the samples were described in detail in Jeong et al. [41]. Briefly, the coal samples were thoroughly dried, weighed to approximately 50 μg, and enclosed in tin capsules for carbon isotope analysis. δ^13^C values were measured using a VisION mass spectrometer (Isoprime, Manchester, United Kingdom) interfaced with a Vario PyroCube elemental analyzer (Elementar, Hesse, Germany) at KBSI. Carbon isotope ratios were expressed relative to the V-PDB standard, using delta notation as shown above. δ^13^C values were normalized using international standards IAEA-600, NBS-22a, USGS-40, and IAEA-CH6 with assigned δ^13^C values of −27.8‰, −29.7‰, −26.4‰, and −10.5‰, respectively, and laboratory standard UREA with assigned δ^13^C values of −35.46‰. The precision of the δ^13^C analysis was assessed through replicate analysis of the three international standard materials. The standard deviation of δ^13^C value for all the standard materials was less than ±0.2‰ (n = 3) for each analytical batch.

**Table 2:** Concentrations and δ^13^C values of CO_2_ (δ^13^C-CO_2_) derived from natural and anthropogenic sources.

Sample type	δ^13^C-CO_2_ (‰)	CO_2_ (ppm)	n	Remark
Exhaust gas (gasoline)	−28.2	–	1	Chevrolet (Malibu)
	−28.1	–	1	Samsung (XM3)
Exhaust gas (diesel)	−25.9	–	1	Volkswargen (Golf)
	−25.3	–	1	Chevrolet (Orlando)
	−25.8	–	1	KIA (K3)
Soil	−25.5 ± 2.2	–	10	
Ambient air in forest	−8.9 ± 0.4	437 ± 8	4	

### Soil samples

Soil samples of 1 kg (n = 10) were collected near areas where the ambient air samples were collected. Samples were carefully collected from the forests to minimize the influence of anthropogenic factors on soil carbon content. To obtain representative soil from each sampling site, soil was collected at a depth of 20 cm from four points approximately 10 m apart and one point in the center and then mixed well. The soil samples were individually packed in Ziploc (25 × 30 cm) bags and transported to the laboratory. After handpicking leaves and litter from the soil samples, they were completely dried at room temperature in the laboratory and sieved to a particle size of 2 mm. The dried samples were soaked in a 10% HCl solution to remove inorganic carbon from the soil samples, washed several times with deionized water, re-dried in an oven maintained at 40 °C, and stored in a vacuum oven until analysis was completed. Similar to the coal samples, the carbon isotope compositions of the soil samples were determined at KBSI. δ^13^C values were normalized using international standards IAEA-600, USGS-40, and IAEA-CH6, respectively, and laboratory standard urea with assigned δ^13^C values of –8.0‰.

## Results and discussion

Table 2 lists CO_2_ concentrations and δ^13^C-CO_2_ values of ambient air in the forest, and δ^13^C values of potential anthropogenic sources (i.e., vehicle exhaust gas, coal combustion) that contribute chemically and isotopically to atmospheric CO_2._ As mentioned earlier, CO_2_ concentrations of these anthropogenic sources could not be directly measured due to their extremely high levels. CO_2_ concentrations and isotopic compositions of atmospheric CO_2_ collected from urban (including tunnels and roadside) and rural areas (inland and coastal areas) are shown in the supplementary S1–S3 Tables.

### Air pollutants: CO_2_ emissions from vehicle exhaust and coal

**Fig 2:**
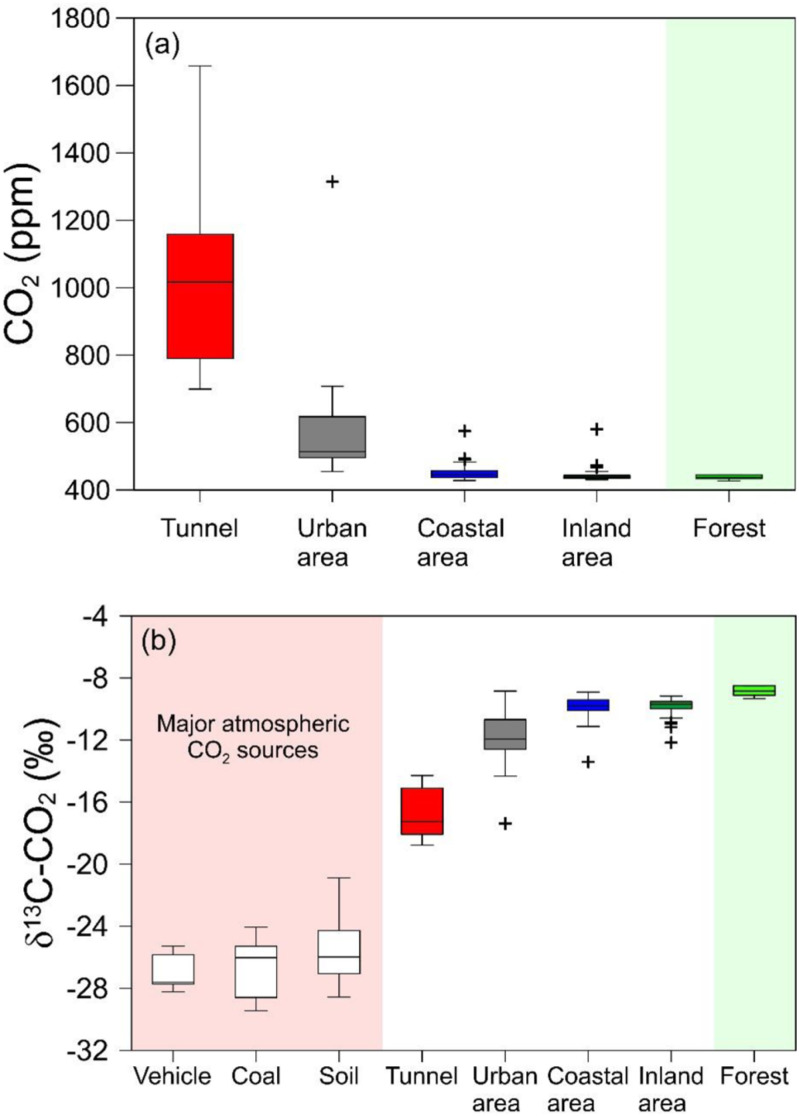
Box-Whisker diagrams showing concentrations (a) and δ^13^**C values (b) for CO**_**2**_ **gas in major natural and anthropogenic sources and air samples collected in the study areas.** The red background represents typical sources of atmospheric CO_2_, while the green background is marked to indicate the forest. The box and central line represent the interquartile range (IQR; Q3–Q1) and the median, respectively. The upper and lower whiskers represent Q3 + 1.5 × IQR and Q1 − 1.5 × IQR, respectively.

As mentioned earlier, the CO_2_ concentration of vehicle exhaust gas collected using the adopted experimental method could not be estimated because of its very high concentration. Previous studies have shown that the concentrations of CO_2_ from vehicle exhaust and CO_2_ from coal combustion range from 1% to 15% and 3% to 15%, respectively [20,21,23,25]. Therefore, CO_2_ concentrations from contaminant sources can be limited to 1%–15%. In this study, vehicle exhaust gas and coal samples showed δ^13^C values ranging from −28.2‰ to −25.3‰ (avg. −26.7 ± 1.4‰, n = 5) and from −28.1‰ to −22.8‰ (avg. −25.4 ± 1.6‰, n = 68) [41], respectively (Fig 2). The δ^13^C values were similar to results in previous studies; i.e., CO_2_ emitted from gasoline engine vehicles has δ^13^C values ranging from −29.3‰ to −24.4‰ [7,22,24], and CO_2_ from diesel engine vehicles ranges from −29.2‰ to −28.6‰ [22]. The δ^13^C values of global coal range from −27.4‰ to −23.5‰ [42–44]. Meanwhile, the carbon isotope composition of CO_2_ derived from coal-fired power plants may include ^13^C-depleted CO_2_ due to fractionation during combustion processes, which is about 1.3‰ lower than that of coal [45]. Considering the δ^13^C values of coals reported in previous studies and the isotope fractionation associated with coal combustion, CO_2_ derived from vehicles and coal are characterized by similar δ^13^C-CO_2_ values. Subsequently, both CO_2_ sources can be used as end-members with δ^13^C-CO_2_ ranging from −28.2 to −25.3‰ and −29.4 to −24.1‰, respectively.

### Natural sources: soil and atmospheric CO_2_

Soil CO_2_ is produced through the decay of soil organic matter (SOM) and plant respiration and is regulated by complex CO_2_ interactions among the hydrosphere, atmosphere, biosphere, and pedosphere [14,46,47]. Soil respiration occurred mainly at shallow depths in the soil profile. Previous studies have shown that soil CO_2_ concentrations released from soil profiles deeper than 30 cm range from 481 to 74,073 ppm with an average of 17,273 ppm [26,27]. The carbon isotope composition of soil CO_2_ is closely related to the type of vegetation (e.g., C3 or C4 plants) because no significant carbon isotope fractionation occurs during SOM decomposition. Diffusion in the soil zone results in δ^13^C-CO_2_ value as high as 4.4‰ compared to that of SOM [48]. The ^13^C-enriched CO_2_ remaining in the soil zone then mixes with atmospheric CO_2_ and reaches a steady state with δ^13^C-CO_2_ value similar to atmospheric CO_2_ (typically, −10 to −8‰) on the soil surface. Meanwhile, for C3 plant, considering isotopic fractionation during CO_2_ diffusion through stomata (~4.4‰) and during carboxylation (~29‰), photosynthetic discrimination (Δδ^13^C) was estimated as follows [49,50]: Δδ^13^C = 4.4 + (29–4.4)*(C_c_/C_a_), where C_c_ and C_a_ represent chloroplast CO_2_ and ambient CO_2_ concentrations, respectively, and typical value of C_c_/C_a_ is ~ 0.55 [50]. Thus, when the mean δ^13^C of the biosphere is about −26‰, δ^13^C value of atmospheric CO_2_ at equilibrium would be approximately −8‰. The concentration and δ^13^C value of atmospheric CO_2_ near the soil surface depend on those of soil CO_2_. In this study, the δ^13^C value for soil samples ranged from −28.6 to −20.9‰ (avg. −25.5 ± 2.2‰), with values ranging from −26.9 to −24.4‰ corresponding to the 25^th^–75^th^ percentile. According to photosynthetic discrimination in previous studies, atmospheric CO_2_ near the soil surface should range from −8.9 to −6.4‰.

Air samples collected from the forests showed a narrow range of CO_2_ concentrations, ranging from 427 to 445 ppm, with an average of 437 ppm. In 2022, this concentration was similar to that (415–434 ppm, avg. 424 ppm) reported at the TAP on the west coast of Taean, South Korea, a NOAA observation site. The carbon isotope composition for the samples from forests and TAP ranged from −9.3‰ to −8.5‰ (avg. −8.9‰) and from −9.5‰ to −8.3‰ (avg. −9.0‰), respectively (data available at https://gml.noaa.gov/dv/iadv/graph.php?code=TAP&program=ccgg&type=ts). Considering that the administration area including TAP has a small population (approximately 9,000 individuals), CO_2_ contribution from anthropogenic sources can be negligible, and both carbon isotope compositions were similar to those estimated for the soil samples collected in this study. Thus, the forest samples used in this study were considered representative of natural sources regarding CO_2_ concentration and carbon isotope composition.

### Air samples

Atmospheric CO_2_ concentrations in urban areas range from 455 to 1,314 ppm (avg. 625 ± 271 ppm), whereas those in rural areas range from 428 to 580 ppm (avg. 449 ± 22 ppm) (Fig 2a). Rural samples from coastal and inland areas had similar CO_2_ concentrations, 450 ± 20 ppm, and 446 ± 26 ppm, respectively. The δ^13^C values of CO_2_ for air samples in urban and rural areas ranged from −17.4 to −8.8‰ (avg. −12.1 ± 2.5‰) and from −13.4 to −8.9‰ (avg. −9.8 ± 0.6‰), respectively (Fig 2b). Like CO_2_ concentration, samples from coastal and inland areas showed similar δ^13^C-CO_2_ values of −9.8 ± 0.6‰ and −9.9 ± 0.6‰, respectively. During sampling, two samples (RC-3 and RI-3) from coastal and inland areas were polluted with vehicle exhaust gas, showing the highest CO_2_ concentrations and lowest δ^13^C-CO_2_ values. Except for these two samples, no temporal variations in CO_2_ concentration and carbon isotope composition were observed.

Air samples collected from the tunnels exhibited relatively high CO_2_ concentrations of 699–1,658 ppm (avg. 1,030 ± 299 ppm) and significantly lower δ^13^C-CO_2_ values of −18.8 to −14.3‰ (avg. −16.9 ± 1.6‰) compared to air samples from urban and rural areas. For tunnel samples collected two or three times (T-1, T-2, T-3, and T-4), CO_2_ concentrations and δ^13^C-CO_2_ values varied significantly during the study period, with δ^13^C-CO_2_ values systematically decreasing as CO_2_ concentrations increased and vice versa. For example, the T-2 sample collected in January 2023 had a CO_2_ concentration up to approximately 2.4 times higher than in October 2022, while δ^13^C-CO_2_ value was −18.4‰ in January 2023 and −14.7‰ in October 2022.

### Sources of CO_2_ in air samples: using the Keeling plots

The ‘Keeling plot’ method was applied to determine the contribution and trends of various sources to atmospheric CO_2_ [51]. When the reciprocal of CO_2_ concentration (1/CO_2_) is plotted against δ^13^C-CO_2_ value, the samples are represented by a binary mixing line of δ^13^C-CO_2_ = a × 1/CO_2 _+ b, which indicates that two major sources determine the CO_2_ concentrations and δ^13^C-CO_2_ values of samples. Atmospheric CO_2_, which includes both natural and anthropogenic contributions, is typically selected as one of the main CO_2_ sources (first endmember). For the second endmember, a pure CO_2_ source, it is theoretically possible to estimate δ^13^C value of that source assuming the observed relationship arises from two-component mixing. Thus, if the mixing line is expressed with a high R-squared value, the CO_2_ samples can be reliably explained by contributions from the two main sources. In contrast, a low R-squared value suggests interference from additional sources or variability in the endmembers.

**Fig 3:**
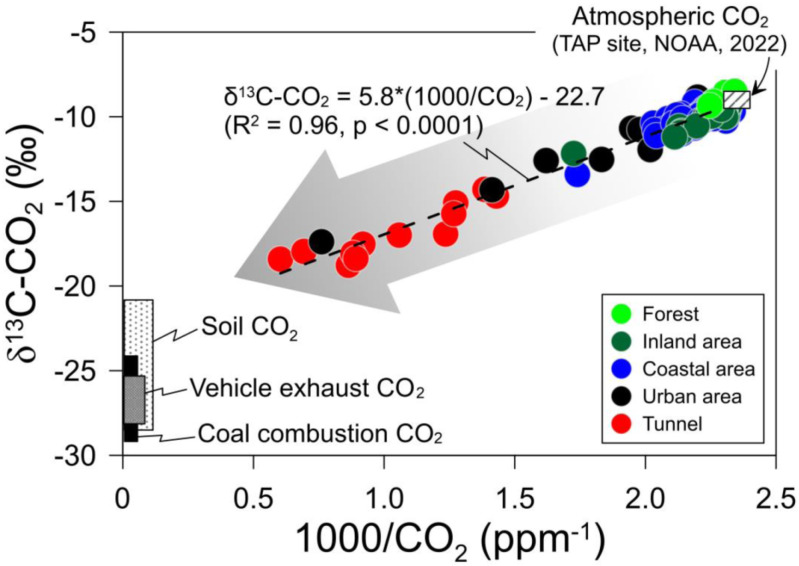
Keeling plot of 1000/CO_2_ and δ^13^**C value for CO**_**2**_ **gas in air samples collected from tunnel, urban and rural areas.** The samples in rural areas were collected from coastal area where coal-fired power plants are located and from inland area.

In this study, the correlation between samples was determined through plots of 1/CO_2_ and δ^13^C-CO_2_ values (Fig 3). Major sources of atmospheric CO_2_ include CO_2_ from soil, vehicle exhaust gas, and coal combustion, which have significantly low δ^13^C values with greatly elevated CO_2_ concentrations. Subsequently, the natural (i.e., soil CO_2_) and anthropogenic sources were displayed on the lower left of the plot, while atmospheric CO_2_ was characterized by a δ^13^C-CO_2_ value of −8.9‰ at a CO_2_ concentration of 437 ppm, located on the upper right. All air samples collected in this study varied between CO_2_ derived from external sources (e.g., vehicle exhaust, coal combustion, and soil CO_2_) and CO_2_ derived from the atmosphere, indicating that the CO_2_ in the samples was formed by mixing atmospheric CO_2_ and CO_2_ derived from external sources.

**Fig 4:**
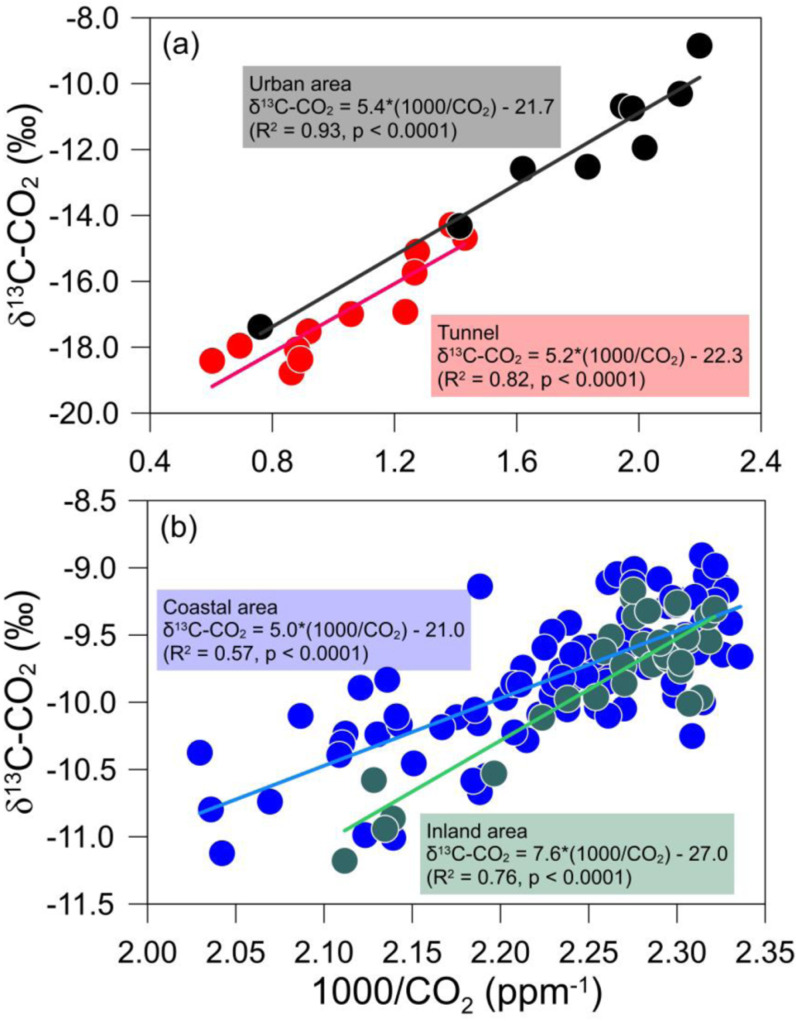
Keeling plots of 1000/CO_2_ and δ^13^C-CO_2_ values for urban area and tunnel samples (a), and air samples from coastal and inland areas (b). There are several coal-fired power plants in the coastal area.

Air samples from the tunnels shifted toward natural and anthropogenic sources in the plot shown in Fig 3, whereas air samples from urban, rural, and forested areas were generally closer to the representative atmospheric CO_2_ range. Some urban samples were distant from atmospheric CO_2_ end-members. To better determine the contribution of sources to ambient air samples, tunnel and urban samples (Fig 4a) and rural samples (Fig 4b) were plotted separately on the plot of 1000/CO_2_ and δ^13^C value. Both the tunnel and urban samples generated regression lines with similar slopes and intercepts: δ^13^C-CO_2_ = 5.2 × 1000/CO_2_–22.3‰ (R² = 0.82) and δ^13^C-CO_2_ = 5.4 × 1000/CO_2_–21.7‰ (R² = 0.93), respectively. These results indicated that the high concentrations of ^13^C-depleted CO_2_ in the urban samples were primarily due to vehicle exhaust gas. This observation suggests that vehicle exhaust gas contributes significantly to increase CO_2_ in urban areas. The poor correlation for tunnel samples would result from differing CO_2_ contribution from gasoline and diesel vehicles. In practice, carbon isotope compositions of CO_2_ emitted from the two vehicle types showed a deviation of approximately 3‰ (Table 2). The slope and intercept of the urban samples were similar to those (δ^13^C-CO_2_ = 4.8 × 1000/CO_2_–22.3‰) reported for polluted CO_2_ in Poland [52]. As traffic volumes increase in urban areas, CO_2_ concentrations increase [53]. Furthermore, urbanization changes land use, reducing the natural spaces available to absorb CO_2_ through photosynthesis and increasing CO_2_ concentrations in urban atmospheres [54]. Unexpectedly, the estimated intercept from the tunnel samples did not shift toward the vehicle exhaust gas on the plot but showed higher δ^13^C-CO_2_ value. This result may reflect the nature of sufficiently polluted atmospheric CO_2_ and/or the small number of samples. Except for the two samples with the highest CO_2_ concentrations, the δ^13^C-CO_2_ value was estimated at −24.3‰.

During the study period, the wind direction in the urban area (center of Seoul) was predominant from the northwest to southeast in the morning and from the southwest to northeast in the afternoon, with wind speeds mostly at ~2m/s [55]. Based on δ^13^C-CO_2_ values of the urban samples in this study, the urban CO_2_ dome was mostly influenced by locally produced CO_2_, regardless of the advected CO_2_ from the northwest and southwest, or vertical mixing during the day. This interpretation is consistent with findings from previous studies. Idso et al. [56] reported that near-surface CO_2_ concentrations in urban areas varied between pre-dawn and mid-afternoon time periods, and those in the mid-afternoon were generally reduced due to enhanced vertical mixing. The loss of CO_2_ due to the vertical mixing was compensated by advection of CO_2_ produced in city and CO_2_ domes were preserved. In a recent study, Di Martino et al. [57] found that airborne CO_2_ in the Naples metropolitan area showed ^13^C-depleted isotopic signature from −17.65‰ to −8.54‰ (average ~ −11‰) due to vehicle exhaust emissions, forming an urban CO_2_ dome. The contribution of the anthropogenic CO_2_ to atmosphere was also diluted by advected CO_2_ from the sea.

In contrast, the coastal and inland samples from the rural areas exhibited different slopes and intercepts (Fig 4b). Samples from the coastal area had a regression line with a lower slope and higher intercept than samples from inland areas, which were similar to the regression lines from urban areas. These results suggest that CO_2_ sources contributing to atmospheric CO_2_ in coastal areas are different from those in inland areas. Based on the slope and intercept, coastal samples are likely to be affected by sources with carbon isotope signatures similar to the CO_2_ sources contributing to the urban samples.

According to population and traffic statistics for coastal areas, the population and traffic volume are relatively small compared to urban areas such as Seoul. Coal-fired power plants have been operational for a long time since the late 1990s [58,59]. The linear regression line for the coastal samples was similar to that for the urban areas; however, the statistics and existence of apparent CO_2_ suppliers suggest that CO_2_ from coal, rather than CO_2_ from vehicle exhaust, maybe a significant source of emissions. CO_2_ concentrations and their carbon isotopic compositions would be affected by varying contributions from different sources depending on the sampling period and time. Nonetheless, the samples from urban, rural and tunnel were explained by the corresponding binary mixing lines on the keeling plot. Meanwhile, air quality in South Korea has been affected by the budget of air pollutants from China owing to westerly winds. The δ^13^C values of CO_2_ gas derived from Chinese coals and coals currently used in South Korea was estimated to be in the range of −30.6 to −23.4‰ and from −29.4 to −24.1‰, respectively when considering isotope fractionation during the process of converting coal into CO_2_ gas. These values were slightly lower than the estimated δ^13^C value for one of the primary sources contributing to CO_2_ in coastal area samples. These results indicate that coal-fired power plants are a major source of air in coastal areas.

Samples from inland areas were represented by a linear regression line with the highest slope and lowest intercept: δ^13^C-CO_2_ (‰) = 7.6 × 1000/CO_2_–27.0 (R² = 0.76). The estimated δ^13^C value for one of the sources was similar to that of soil CO_2_ in this study, considering carbon isotope fractionation due to diffusion of soil-respired CO_2_ is 4.4‰ [60]. In addition, according to Shin et al. [61], the δ^13^C values of organic materials (e.g., leaf litters) ranged from −31.1 to −29.3‰, resulting in δ^13^C values of approximately −27‰. The estimated δ^13^C value from inland samples differed clearly from coastal samples characterized by similar population and traffic levels. In addition, the slopes of the coastal and inland samples were distinguished. Compared to the land-use patterns at other sampling sites, inland areas were collected from regions with a higher proportion of forests. This result indicated that the major CO_2_ source in the inland samples was controlled by a combination of soil CO_2_ and atmospheric CO_2_. In previous studies, uncontaminated air samples were located between soil CO_2_ and atmospheric CO_2_ end-members on the Keeling plot, represented by a linear regression line with a slope of 8.3 and an intercept of −29.6‰ [22]. Note that decrease of tree-ring δ^13^C values for some decades was induced by rising levels of CO_2_ mainly due to massive vehicle exhaust emissions [62]. The influence of photosynthetic CO_2_ uptake on ambient CO_2_ concentration and δ^13^C value would also be reflected in the inland samples.

## Conclusions

A total of 151 air samples were collected from urban and rural areas and analyzed for atmospheric CO_2_ concentration and carbon isotopic composition. We identified the major sources of CO_2_ by considering land-use patterns. Representative CO_2_ sources were collected during the sampling campaigns. The CO_2_ concentrations were higher in urban areas than in rural areas. In rural areas where coal-fired power plants are located, and forests dominate, CO_2_ concentrations were similar regardless of land-use patterns. The deviation between urban and rural samples and the similarity among rural samples were associated with δ^13^C values. Urban samples have ^13^C-depleted CO_2_ in the range of −17.4 to −8.8‰ (avg. −12.1 ± 2.5‰), while rural samples showed relatively higher δ^13^C values of −13.4 to −8.9‰ (avg. −9.8 ± 0.6‰). Meanwhile, the urban samples showed similar CO_2_ concentrations and δ^13^C-CO_2_ values to the tunnel samples. On a plot of 1/CO_2_ and δ^13^C-CO_2_ showing representative CO_2_ sources, urban and tunnel samples had similar slopes and intercepts, shifting toward vehicle exhaust gas, indicating that air quality in urban areas is mainly affected by contaminant sources such as fossil fuel emissions. Among the rural samples, those collected near coal-fired power plants had slopes and intercepts different from those of other rural samples and were quite similar to those of urban samples. These results indicate that emissions (including CO_2_) from coal-fired power plants affect air quality in rural areas. In contrast, rural samples from forest-dominated areas showed the highest slope and lowest intercept, indicating that natural sources such as soil CO_2_ play a major role in determining air quality in rural areas. The relationship between atmospheric CO_2_ concentration and δ^13^C-CO_2_ values can be used to identify specific regions and distinguish CO_2_ sources. However, distinguishing sources with overlapping carbon isotope compositions requires additional information and analytical techniques.

## Supporting information

S1 Table Averages of CO_2_ concentrations and δ^13^C-CO_2_ values in air samples collected from roadside and tunnels in urban areas. (XLSX)

S2 Table Concentrations and δ^13^C-CO_2_ values of CO_2_ in air samples collected from rural sites located near coastal area. (XLSX)

S3 Table Averages of CO_2_ concentrations and δ^13^C-CO_2_ values in air samples collected from rural-inland area. (XLSX)
